# Sex May Modulate the Effects of Combined Polyphenol Extract and L-citrulline Supplementation on Ambulatory Blood Pressure in Adults with Prehypertension: A Randomized Controlled Trial

**DOI:** 10.3390/nu13020399

**Published:** 2021-01-27

**Authors:** Cécile Vors, Maryka Rancourt-Bouchard, Charles Couillard, Iris Gigleux, Patrick Couture, Benoît Lamarche

**Affiliations:** 1 Centre Nutrition, Santé et Société (NUTRISS), Institut sur la Nutrition et les Aliments Fonctionnels (INAF), Université Laval, Québec, QC G1V 0A6, Canada; maryka.rancourt-bouchard@fsaa.ulaval.ca (M.R.-B.); Charles.Couillard@fsaa.ulaval.ca (C.C.); Iris.Gigleux@fsaa.ulaval.ca (I.G.); patrick.couture@crchul.ulaval.ca (P.C.); Benoit.Lamarche@fsaa.ulaval.ca (B.L.); 2 CHU de Québec Research Center, Université Laval, Québec, QC G1V 0A6, Canada

**Keywords:** polyphenols, grape seeds, cranberries, L-citrulline, antioxidants, hypertension, cardiovascular diseases, glycation end products, endothelial function

## Abstract

Increased blood pressure (BP), vascular dysfunction and inflammation are involved in the etiology of cardiovascular disease (CVD). Although several dietary components such as polyphenols and L-citrulline may help to control BP, their combined impact on ambulatory BP in individuals at risk of CVD remains unknown. The objective of this research was to investigate the short-term impact of supplementation with a combination of polyphenol extract and L-citrulline on ambulatory BP, endothelial function and inflammation. In a randomized double-blind parallel trial, 73 men and women with prehypertension were supplemented with a placebo (cellulose, *n* = 34, Plac) or 548 mg/day of polyphenols and 2 g/day of L-citrulline (*n* = 35, Suppl) for 6 weeks. The primary outcome of this study was the difference between groups in 24-h ambulatory diastolic BP (DBP) at week six. Secondary outcomes were a difference between groups at week six in ambulatory systolic BP (SBP), casual BP, serum lipids and high-sensitivity C-reactive protein (hs-CRP) concentrations and skin advanced glycation end products (AGEs). Potential interaction of treatment with sex was examined. Suppl had no impact on mean ambulatory SBP and DBP (*p* > 0.10 vs. placebo). Daytime and 24-h SBP were reduced with Suppl in women (*p* ≤ 0.01), but not in men (*p* ≥ 0.27). A non-significant reduction in AGEs was observed after Suppl compared to Plac among all participants (*p* = 0.07) and there was no difference in the concentrations of blood lipids (*p* > 0.20) or CRP (*p* = 0.36) between treatments at week six. Therefore, supplementation with polyphenol extract and L-citrulline for 6 weeks has no impact on ambulatory BP, blood lipids and CRP in adults with prehypertension. However, the polyphenol extract/L-citrulline supplement may reduce ambulatory SBP in women, but not in men. These preliminary results need further research efforts towards further documenting this sex-dependent BP response to supplementation with polyphenols and L-citrulline.

## 1. Introduction

Hypertension is a causal risk factor for a variety of cardiovascular diseases (CVDs) [1]. Treatment of high blood pressure is one of the cornerstones of CVD prevention, even among individuals with prehypertension who are at increased risk for progression to hypertension. Indeed, men and women in the 130/80 to 139/89 mmHg blood pressure (BP) range are at twice the risk of developing hypertension compared to those with BPs <130/80 mmHg [2]. No anti-hypertensive drugs are indicated to treat prehypertension and lifestyle modifications are strongly recommended [3,4]. Indeed, substantial evidence suggests that diet and lifestyle play an important role in BP control [5,6,7,8] and adoption of healthy dietary habits by individuals with prehypertension remains the cornerstone therapy.

Ambulatory BP monitoring (ABPM) is a proven and reliable method to assess BP according to hypertension guidelines around the world [4]. Recent studies suggest that ABPM data more accurately reflect an individual’s actual BP than casual or in-office BP measurements [9,10]. Moreover, several lines of evidence indicate that ambulatory BP monitoring data correlate more closely with target organ injury [3] and CVD risk [11] than conventional in-office measurements. To date, very few nutritional intervention studies have used 24-h ABPM to assess nutrient/food and diet efficacy in modifying BP [12,13,14,15,16].

Besides hypertension, vascular endothelial dysfunction and inflammation also play key etiological roles in the CVD pathophysiology. Vascular endothelial cells express and secrete a large selection of molecules, including adhesion molecules, which are involved in the modulation of thrombosis and inflammation [17]. Chronic activation of the vascular endothelium plays an integral role in the development of atherosclerosis and CVD [18,19]. Advanced glycation end products (AGEs) are known to modulate vascular function and produce vascular damage [20]. AGEs can interact with compounds in the blood vessel wall to alter their function [21]. AGEs can also ligate to specific receptors present on endothelial cells [21], causing the release of intercellular mediators with pro-inflammatory and pro-fibrotic effects, and resulting in the development of vascular endothelial dysfunction [22].

Data from randomized controlled trials (RCTs) suggest that supplementation with polyphenols from a variety of food sources may reduce inflammation (*n* = 118) [23,24,25] as well as blood pressure, particularly among individuals with higher usual BP (*n* = 236) [25,26,27,28]. L-citrulline is a non-essential amino acid naturally present in many foods (dairy, meats, seafood, grains, vegetables and nuts) and is found in large amounts in watermelon. L-citrulline stimulates the production of nitric oxide (NO), a key molecule in the vasculature that regulates vascular tone and blood flow [29], via de novo L-arginine production [30,31]. L-citrulline may, thus, improve vascular function through increased L-arginine bioavailability and nitric oxide synthesis [30,32]. Recent studies have shown that chronic L-citrulline supplementation may decrease usual blood pressure [33,34]. However, no clinical study has yet investigated the impact of the promising combination of polyphenol and L-citrulline supplementation on ambulatory BP and other cardiometabolic risk markers.

The general objective of the present study was to investigate the effects of short-term supplementation with a combination of polyphenols from cranberry and grape seed extracts and L-citrulline on 24-h ambulatory BP and markers of endothelial function and inflammation in men and women with prehypertension compared to a placebo. The primary outcome of this RCT was the difference between groups in 24-h ambulatory DBP at week six (post-treatment values). Secondary outcomes were differences between groups in 24-h ambulatory SBP, blood lipids concentrations, skin levels of glycation end products and blood concentrations of CRP (C-reactive protein) at week six. We hypothesized that a supplementation combining polyphenol extracts and L-citrulline may significantly reduce 24-h ambulatory BP and associated cardiometabolic markers in individuals with prehypertension.

## 2. Materials and Methods

### 2.1. Participants

Men and women were recruited in Quebec City primarily through mailing lists and announcements published on our website. Participants had to be aged between 18 and 75 years and have maintained a stable body weight (±2.5 kg) for at least 3 months prior to the study. All participants underwent an array of measurements and answered a medical history questionnaire at screening. The a priori defined eligibility criterion was to have a mean daytime ambulatory SBP ≥125 mmHg and <135 mmHg and a mean daytime ambulatory DBP <85 mmHg. However, this criterion was modified 2 months into recruitment due to difficulties in achieving the intended sample size (only 13% of individuals had met these criteria). Eligibility based on mean daytime ambulatory SBP values was changed to ≥120 mmHg and <140 mmHg and mean daytime ambulatory DBP to <90 mmHg. Exclusion criteria were a history of CVD, type 2 diabetes or monogenic dyslipidemia; uncontrolled endocrine disorder; kidney stones; any clinical signs or laboratory evidence for inflammatory, gastrointestinal, endocrine, renal, pulmonary, neurological, cardiovascular, metabolic or hematological problems or cancer; the use of anticoagulants or thrombocyte aggregation inhibitors, chemotherapeutic agents, anti-inflammatory drugs, medication for blood lipids, diabetes, hypertension, erectile dysfunction or auto-immune diseases; alcohol consumption >14 consumption/week (i.e., >188 g ethanol/week); drug consumption; hypersensitivity or allergy to one of the ingredients in polyphenols/L-citrulline supplement or placebo; use of additional cranberry or grape seed extract or citrulline products; women who were breastfeeding, pregnant or planning a pregnancy; and women in perimenopause. Women with child-bearing potential had to use one of the following contraceptive methods during the study: hormonal birth control methods, intrauterine devices, confirmed successful vasectomy of partner or total abstinence. Pregnancy test was also performed by measuring plasma level of beta human chorionic gonadotropin at screening. Postmenopausal women who did not have regular menses ≥1 year were eligible. However, hormone supplementation status had to be constant for at least 6 months prior to the study and for the duration of the study. When a perimenopausal status was suspected, follicle-stimulating hormone measurements were performed. The use of an anti-inflammatory drug was prohibited during the intervention. Other medications, vitamin and mineral supplements and natural health products were allowed under the approval of the study physician as long as the use and dosage were stable before and throughout the study period. The period of stability before the intervention depended on the type of drug or supplement: anti-inflammatory drugs and antibiotics had to be stopped 10 days before the first day of intervention (D0); vitamins and minerals had to be stable for more than 2 weeks; omega 3 for 1 month; mental health drugs and hormonal contraception should have been stable for more than 3 months and for replacement hormones, 6 months.

The study protocol was approved by local Research Ethics Committees (2017-207) and was fully explained to all participants, who gave their written informed consent before participation. The study protocol is registered at ClinicalTrials.gov, NCT03679195.

### 2.2. Study Design

The study was undertaken according to a double-blind, 1:1 randomized, parallel, placebo-controlled design and was conducted between September 2018 and June 2019 at the Institute of Nutrition and Functional Foods (INAF) in Quebec City, Canada. A total of 73 participants who met all inclusion criteria were randomly assigned to a 6-week period of supplementation with polyphenol extract and L-citrulline or a placebo with the use of an in-house computer program. The randomization was stratified by sex with block sizes of 4. Allocations to treatments were coded and concealed from participants, study coordinators and laboratory technicians throughout the study. The envelope containing the codes was unsealed once all primary statistical analyses had been completed.

### 2.3. Study Supplementation

The supplements of the polyphenol extract and L-citrulline (Suppl) as well as the placebo (Plac) were ingested in the form of capsules of identical appearance, both supplied by Pure Encapsulations (Sudbury, MA, USA). According to the Product License, it is recommended to take 2 capsules daily between meals. In the framework of this pilot study, in participants with prehypertension but who are otherwise healthy, we tested the intake of 4 capsules/day in order to maximize the impact. Participants in the Suppl group were supplemented with 764 mg/day of polyphenol total extract (including 548 mg polyphenols per se) and 2 g/day of L-citrulline. The polyphenol extract in the supplement originated from a proprietary blend called CranLoad™ providing cranberry (*Vaccinium macrocarpon*) extract and grape (*Vitis vinifera*) extract. While achieving intake of a similar amount of polyphenols from food is possible, the same is not true for L-citrulline. One would need to eat approximately 125 g of sweet, dried cranberries to achieve an intake of 548 mg of total phenolic compounds [35], whereas one would have to eat 2 kg of watermelon to achieve an intake of 2 g of L-citrulline. The proportion of all polyphenols in the supplement was 16% from the cranberry extract and 84% from the grape seed extract. In the placebo capsules, the active ingredients were replaced by cellulose.

Participants were instructed to take 2 capsules daily between breakfast and lunch and 2 capsules between lunch and dinner. Capsules had to be taken with water at least 1 h after the preceding meal and 1 h before the next meal. Participants were asked to maintain their usual dietary habits and physical activity level in order to maintain a constant body weight throughout the study. Alcohol consumption ≤14 consumptions/week (i.e., ≤188 g ethanol/week) was allowed during the study. However, participants had to refrain from alcohol 2 days before blood sampling and from vigorous physical activity 1 day before blood sampling.

### 2.4. Compliance

Participants were asked to complete a checklist for supplement consumption and to return their leftover supplements at the end of the intervention. Compliance to supplementation was determined by counting capsules returned to the study coordinator.

### 2.5. 24-h Recall and Physical Activity Diary Record

Dietary intakes were measured using a validated web-based 24-h food recall on 3 random days prior to and in the last week of the intervention. The 24-h food recalls allowed to estimate the energy intake of the participants and also to calculate Canadian Healthy Eating Index (C-HEI) scores, an indicator of overall diet quality, ranging from 0 to 100. Physical activity was monitored before and at the end of the intervention with the use of a 3-day validated physical activity journal. Total daily physical activity (in metabolic equivalent of tasks, METs) was calculated by averaging values from the 3 days.

### 2.6. Risk Factor Assessment

#### 2.6.1. Anthropometry

Body weight and waist and hip circumferences were measured by the same study coordinator throughout the study, according to standardized procedures [36].

#### 2.6.2. Ambulatory BP Monitoring and Office BP Measurement

Twenty-four hour ambulatory BP monitoring and office BP measurements were performed at screening and at the beginning and end of the intervention. Ambulatory BP was monitored on the participant’s non-dominant arm using a Spacelabs 90207 monitor (Spacelabs Inc., Redmond, WA, USA). The daytime period was set from 6:00 to 22:00, during which BP was measured every 20 min. BP was measured every hour during the nighttime period. Participants were asked to report the dominant physical activity performed for each 15-min block of the BP monitoring period to assess daily energy expenditure [37]. They were asked to refrain from vigorous physical activity during the entire monitoring period. To be considered valid and reliable, ≥70% of successful BP readings were required for each ambulatory BP monitoring session, as recommended [38]. Office BP was measured in the fasting state by trained research staff at our Clinical Investigation Unit using a calibrated, automatic BP monitor (Digital BPM HEM-907XL Thru, Omron Healthcare, Lake Forest, IL, USA). Participants remained seated for 10 min, after which 3 sequential readings were taken at 3-min intervals.

#### 2.6.3. Advanced Glycation End Products (AGEs)

Accumulation of AGEs in participants’ skin was estimated by autofluorescence at the beginning and end of the intervention, using an AGE Scanner Mini (Diagnoptics, Groningen, The Netherlands) [39]. The AGE Scanner Mini is a variant of the AGE Reader technology, through which skin autofluorescence is calculated as the ratio between the total emission intensity (420–600 nm) and the total excitation intensity (300–420 nm), multiplied by 100, and is expressed in arbitrary units [40]. The optical system used in the AGE Scanner Mini is the same as in the AGE Reader, and the AGE Scanner Mini results correspond to the AGE Reader results multiplied by 100 (according to the manufacturer). Three sequential measurements were taken on a clean and homogeneous area of skin on the participant’s dominant forearm and used as a surrogate marker of endothelial function [41,42,43,44,45].

#### 2.6.4. Cardiometabolic Risk Factors

Blood samples were collected after a 12-h fast at screening and at the beginning and end of the intervention for the measurement of serum total cholesterol, triglyceride and high-density lipoprotein (HDL) cholesterol concentrations using a Dimension Vista 1500 System (Siemens Healthcare Diagnostics, Newmark, DE, USA). LDL cholesterol concentrations were calculated with the Friedewald equation [46]. Serum high-sensitivity C-reactive protein (hs-CRP) concentrations were measured using a BN ProSpec System (Siemens). Hs-CRP values >10 mg/L were considered as missing in the analyses (*n* = 3 samples during the entire study). Fasting blood glucose concentrations were measured once at screening, using a Dimension Vista 1500 system (Siemens, Erlangen, Germany).

### 2.7. Sample Size Calculation

The difference in 24-h ambulatory DBP between values measured after polyphenol extract and L-citrulline and placebo supplementations was the a priori defined primary outcome and was used for sample-size calculations. Sample size was estimated based on data from previous studies [12,13,14,15,16], reporting a change in 24-h ambulatory DBP between different nutritional interventions. The averaged standard deviation (SD) of the change in 24-h ambulatory DBP was 5.1 mmHg. It was determined that *n* = 66 individuals would allow for detecting a clinically meaningful 3.6-mmHg difference in 24-h ambulatory DBP between treatments with a power of 80% and a two-sided type 1 error (α) of 5%, based on the SD of 5.1 mmHg. Based on a projected dropout rate of 10%, the required sample size was *n* = 73. A priori defined secondary outcomes were 24-h systolic blood pressure, blood lipids, markers of vascular function (AGEs) and inflammation biomarkers. Analysis of the potential interaction of treatment with sex was considered exploratory.

### 2.8. Statistical Analyses

Data analysis was performed using SAS statistical software version 9.4 (SAS Institute Inc., Cary, NC, USA). Compliance to supplementation was compared between treatments using the Kruskal–Wallis test. Study outcomes were compared between treatments using post-treatment values and the GENMOD (generalized linear model) procedure. Using a parsimonious modeling approach, baseline values of the selected outcome (measured at screening), sex, age, body weight, waist circumference, physical activity and estimated total energy expenditure during the ambulatory BP monitoring were retained as covariables in the final model only when significant (*p* ≤ 0.05). These covariables were selected to address confounding based on their known association with blood pressure. The skewness in the distribution and the model’s residual normality for each study outcome were considered and data were log-transformed when required. In exploratory analyses, potential interactions of treatment with baseline values of the selected outcome, sex and waist circumference were examined. Data were analyzed on a per protocol basis. There were missing values only for CRP (3 samples) and for nighttime ambulatory BP analysis (1 sample due to unreliable data). Because the GENMOD procedure is robust to missing data, analyses were conducted without the multiple imputation of missing data. As indicated above, all statistical analyses were undertaken while being blinded to treatment allocation.

## 3. Results

### 3.1. Participant Characteristics at Baseline

**Figure 1:**
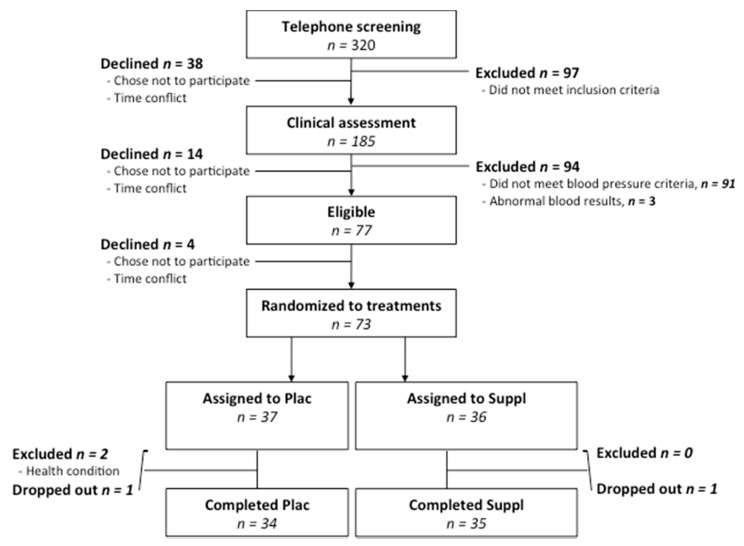
Flow diagram of study participants with prehypertension randomly assigned to a placebo (Plac) or a polyphenol extract and L-citrulline supplement (Suppl).

Figure 1 represents a flow diagram of the study. From a total of 185 men and women screened, 77 were eligible and 73 participants were randomly assigned to one of the two study treatments: 36 to the Suppl group and 37 to the Plac group. Table 1 shows the baseline characteristics for each group. The study included 42 women (58%) and the mean age of participants was 49 ± 19 years. The mean daytime ambulatory SBP at screening was 128.6 ± 5.6 mmHg. All the participants were otherwise healthy. The dropout rate was 5.5% (*n* = 4): two participants dropped out because of protocol-related adverse effects (one in the Plac group had difficulty producing stools, and one in the Suppl group had gastroesophageal reflux) and two participants in the Plac group were excluded from the analyses due to health problems not related to the study treatments.

### 3.2. Compliance to Treatments and Adverse Effects

Based on the returned capsules, the mean compliance to supplementation was high and similar between treatments (Plac group, 96.1% ± 8.8%; Suppl group, 97.3% ± 4.6%; Kruskal–Wallis test: *p* = 0.81). In sensitivity analyses, excluding participants with compliance <80% *(n* = 2, both in Plac group) led to similar results (not shown). There was no difference in the frequency of self-reported adverse effects between treatments (not shown).

### 3.3. Dietary Intake and Physical Activity

There was no difference between groups in physical activity, in energy intake and in overall diet quality at baseline (not shown). There was also no change in any of these variables during the intervention (not shown).

### 3.4. Use of Supplements and Drugs during the Study

Although participants were asked not to initiate or stop taking a drug or supplement during the study, there were some changes as shown in Table S1. Eleven participants had a change in medication during the study (anti-histamine (*n* = 4); anti-inflammatory (*n* = 2) (more than 10 days before the end of the intervention); inhaled anti-inflammatory (*n* = 2); antibiotic (*n* = 2); flu vaccine (*n* = 1)). Table S2 shows the type of drug and corresponding dose taken by participants under stable medication and the change in medication during the intervention. These medications have little effect on blood pressure. Five volunteers had a change (starting or stopping intake) in supplements (natural health products, NHPs) during the study (Supplemental Table S1). Supplements were omega 3, multivitamin, calcium, vitamin B complex, vitamin C, vitamin D, vitamin E, Genacol, vitamin B12, spirulina, curcuma, glucosamine, lutein, collagen, melatonin, probiotics, iron, echinacea, creatine supplementation and, branched-chain amino acids. Most of these supplements have no known effect on blood pressure.

### 3.5. Anthropometry

There was no difference between groups in post-treatment body weight (*p* = 0.33), body mass index (*p* = 0.28) and waist circumference (*p* = 0.70) (Table 2).

### 3.6. Ambulatory and Office BP

**Figure 2:**
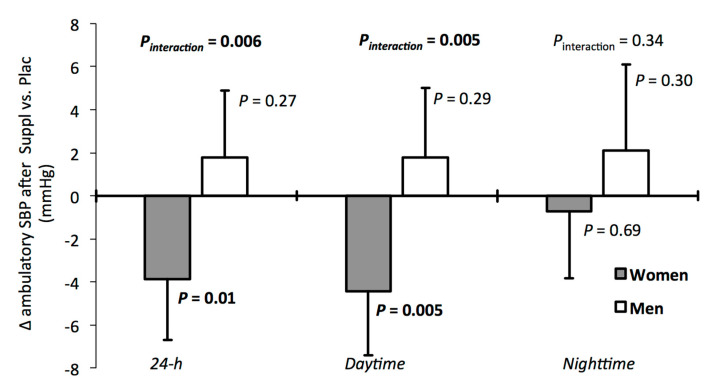
Changes in ambulatory systolic blood pressure (SBP) in response to the polyphenol extract and L-citrulline supplement (Suppl) compared to placebo (Plac) according to sex. Results are shown as differences in least-squares means (Suppl vs. Plac) with 95% CIs obtained from the GENMOD procedure. For men and women, respectively: Plac, *n* = 14 and *n* = 20; Suppl, *n* = 16 and *n* = 19. SBP, systolic blood pressure.

Compared with Plac, Suppl had no impact on mean ambulatory 24-h, daytime and nighttime SBP and DBP (Table 2). However, the exploratory analyses revealed a significant treatment–sex interaction for the change in ambulatory 24-h and daytime SBP (*P*_interaction_ < 0.01 for both, Figure 2), but not for the change in nighttime SBP (*P*_interaction_ = 0.34). Specifically, the ambulatory SBP after Suppl was reduced compared with Plac in women (24-h SBP: −3.9 mmHg (95%CI −6.7, −1.1); daytime SBP: −4.4 mmHg (−7.4, −1.5), Figure 2) but not in men (24-h SBP; +1.8 mmHg (−1.3,4.9); daytime SBP: +1.8 mmHg (−1.5, 5.0), Figure 2). No difference in office SBP (*p* = 0.12) and DBP (*p* = 0.16) was observed between treatments (Table 2).

### 3.7. Blood Lipids and CRP

There was no difference at 6 weeks in the serum concentrations of triglycerides (*p* = 0.30), total cholesterol (*p* = 0.20), HDL cholesterol (*p* = 0.49) and LDL cholesterol (*p* = 0.36) between treatments (Table 2). Plasma levels of hs-CRP were also similar post-treatment between the Suppl and the Plac groups (*p* = 0.36, Table 2). There were no significant treatment–sex interactions in the cardiometabolic response to treatment.

### 3.8. Skin Advanced Glycation End Products (AGEs)

A non-significant reduction in skin AGEs was observed after Suppl compared to Plac supplementation (*p* = 0.07, Table 2). There was no significant treatment–sex interaction in the response of AGEs to treatment.

## 4. Discussion

This study investigated, for the first time, the combined impact of a polyphenol extract and L-citrulline on ambulatory BP in adults with prehypertension as well as on cutaneous AGEs, serum lipids and CRP levels. Despite no mean effect in the entire study sample, the data revealed an interesting sex-dependent response of ambulatory systolic BP to polyphenol extract/L-citrulline supplementation, with women being more responsive than men. There were no significant short-term effects of polyphenol/L-citrulline supplementation on blood lipids, CRP and AGEs in individuals with prehypertension.

Previous clinical studies have suggested that supplementation with polyphenols, especially those isolated from grape seeds, has BP-lowering effects. In a double-blind 8-week RCT among participants with prehypertension and stage 1 hypertension, supplementation with 300 mg/day of grape seed extract reduced office SBP by −5.2 mmHg and DBP by −2.5 mmHg [26]. However, these changes were statistically similar to those observed in the placebo group. Consumption for 4 weeks of a polyphenol-rich grape wine extract containing 800 mg polyphenols in an RCT among untreated individuals with mild hypertension lowered office SBP by −3.0 mmHg and DBP by −1.9 mmHg, but this time, the reductions were greater than those seen in the placebo group [27]. In another recent RCT, supplementing participants with prehypertension and mild hypertension for 12 weeks with 200 mg/day of red grape cell powder, but not 400 mg/day, reduced office DBP compared to a placebo [28]. BP-lowering effects of polyphenols from cranberries have also been shown in a double-blind placebo-controlled parallel-arm study, in which a significant reduction in DBP was observed among individuals consuming low-calorie cranberry juice (173 mg phenolic compounds) daily for 8 weeks compared to individuals consuming the placebo beverage (62 mg phenolic compounds) [25].

Experimental data also suggest potential BP-lowering effects of L-citrulline. Oral L-citrulline is absorbed through the enterocytes and transferred to the portal vein to reach not only the liver (urea cycle) but also the bloodstream, where it is transported, notably, to the kidneys and converted into L-arginine, a key substrate for NO biosynthesis [47]. L-citrulline thus increases NO production indirectly by increasing L-arginine synthesis, which, in turn, may upregulate endothelial vasodilator function and reduce BP. In a recent double-blind RCT, consumption of 6 g/day of watermelon extract rich in L-citrulline for 6 weeks significantly reduced office SBP and DBP (−11.8 and −6.9 mmHg, respectively) in middle-aged individuals with prehypertension and hypertension but showed no differences compared to the placebo group [33]. Short-term 8-week supplementation with 6 g/day of L-citrulline has also been shown to decrease office brachial SBP and DBP (−7.0 and −3.0 mmHg, respectively) and aortic SBP and DBP (−9.0 and −3.0, respectively) in postmenopausal women with prehypertension/hypertension and obesity, but the BP changes were similar to those observed in the placebo group [34]. Surprisingly, the combination of a polyphenol extract with L-citrulline in the present study did not reduce ambulatory BP or office BP in the entire study sample. Differences in methodology, particularly the form, source and dose of the supplements used, may explain the discrepancies among the studies. Indeed, a recent meta-analysis reported that a significant reduction in diastolic BP was observed in response to L-citrulline supplementation only in the studies that used doses ≥6 g/day [48]. Besides, one cannot exclude the possibility that a longer study duration or higher dosages may have led to larger effect sizes on ambulatory blood pressure.

However, the data from the present study suggest that women may be more responsive than men to the systolic BP-lowering effects of a combination of polyphenols/L-citrulline. It is well established that sex hormones play an important role in BP regulation [49]. Estrogens are antihypertensive hormones that cause vasodilation and reduce BP by promoting the activation of endothelial nitric oxide (NO) synthase/NO signaling and by downregulating angiotensin II (AngII) type 1 receptors and endothelin 1 (ET1) receptors [49]. NO is a key molecule in vascular homeostasis through regulation of vascular tone and blood flow, whereas AngII and ET1 are known as potent vasoconstrictors [50,51]. Evidence from animal and human studies has suggested that the BP-lowering effects of polyphenols, regardless of type and source, are NO-dependent [52]. Combined action of polyphenols and estrogens through NO activation may have amplified the reduction in ambulatory SBP among women in the present study. This does not exclude the potential contribution of other mechanisms, considering that postmenopausal women were included in this study (roughly the same number in each treatment group). A recent study reported sex-related differences in the effects of cacao polyphenols on BP in individuals with pre-diabetes [53], with significant reductions in both SBP and DBP seen in women only, who were all postmenopausal [53]. Of note, the reductions in BP after supplementation with the cacao polyphenol extracts were not different than those seen in the placebo group. Interestingly, Odai et al. [54] showed very recently that supplementation with 400 mg grape seed proanthocyanidin extract for 12 weeks did not modify SBP and DBP values in middle-aged Japanese men and women with prehypertension compared with a placebo. Unfortunately, sex differences in the response to treatment could not be explored due to the small sample size (*n* = 10 per group) and the unbalanced sex ratio in study (6 men, 24 women). To the best of our knowledge, no study has yet documented a sex-related difference in BP response to L-citrulline supplementation. L-citrulline may have contributed to the lowering of ambulatory SBP observed in women through its indirect action on NO synthesis, but this needs further investigation.

Hypertension is strongly correlated and often found co-segregating with vascular endothelial dysfunction and inflammation among individuals at risk of CVD [55,56,57]. Increased levels of AGEs have been associated with the progression of diabetes or cardiovascular disorders and AGEs may be a predictive marker for the micro- and macrovascular complications occurring in such diseases [41,58,59]. AGEs accumulate in tissues with slow turnover such as vessel walls and skin and could, thus, be markers of the “metabolic memory” of the body. Owing to the autofluorescent properties of some AGEs, their accumulation within the skin can be easily measured by a non-invasive method reading skin autofluorescence. In the present study, supplementation with polyphenols and L-citrulline led to a non-significant reduction in skin AGEs in individuals with prehypertension compared to those treated with a placebo. However, polyphenols/L-citrulline supplementation had no impact on plasma hs-CRP levels compared with a placebo. Participants presented normal-range skin AGE levels according to their age (mean baseline AGEs 200 ± 53 AU and 202 ± 60 AU in placebo and supplementation groups, respectively) [40] as well as rather normal serum CRP concentrations at baseline (mean CRP 1.5 ± 1.8 and 1.2 ± 1.2 mg/L in placebo and supplementation groups, respectively). The absence of a significant effect of supplementation on cardiometabolic risk factors, including hs-CRP, may in fact be due to the relatively normal cardiometabolic profile of participants in both groups at baseline rather than the study duration. Indeed, study duration has not been shown to modify the impact of anthocyanin supplementation on hs-CRP according to a recent meta-analysis [60]. The extent to which combined supplementation with polyphenols and L-citrulline may have more potent effects on these markers among individuals with more pronounced metabolic disorders (low-grade inflammation, oxidative stress and pre-diabetes) remains to be documented.

The present study has limitations and strengths that need to be outlined. The possibility that L-citrulline may have interfered with polyphenol bioavailability and metabolism, and vice versa, due to additive or antagonistic interactions cannot be excluded. Unfortunately, analysis of polyphenol metabolites after intestinal absorption and transformations was not performed to assess this possibility. Lack of assessment of any changes in plasma NO concentrations and also in plasma markers of oxidative stress and of endothelial function such as plasma AGEs and plasma adhesion molecules is a limitation. Such information may have provided further insights into the mechanisms underlying the ambulatory BP-lowering effects seen in women after supplementation with polyphenols/L-citrulline. Furthermore, testing the two individual extracts might have provided interesting information on their individual effects. However, there are already several papers on each of these supplements and the intent of this research was not to compare the two but simply to assess their combined effect on blood pressure regulation. The present RCT included both women and men, making the results more generalizable to a broader population. Finally and foremost, the BP response to supplementation was assessed using ambulatory BP monitoring, the gold standard for BP measurement.

## 5. Conclusions

The present work shows that supplementation combining a polyphenol extract and L-citrulline for 6 weeks has no impact on ambulatory BP and other cardiometabolic risk markers in adults with prehypertension. However, the polyphenol extract and L-citrulline supplement may reduce ambulatory SBP in women, but not in men. The present findings support future research efforts towards further documenting this sex-dependent BP response to supplementation with polyphenols and L-citrulline.

## Figures and Tables

**Table 1:** Characteristics at screening of all participants with prehypertension randomly assigned a placebo (Plac) or a polyphenol extract and L-citrulline supplement (Suppl) (*n* = 73) ^1^.

	Plac		Suppl	
	*n* = 37		*n =* 36	
Women:men, *n*	22:15		20:16	
Age, year	50.0	±17.5	48.5	±19.8
Body weight, kg	73.3	±16.3	71.8	±12.5
BMI, kg/m^2^	25.9	±5.0	25.3	±3.6
Waist circumference, cm	84.6	±14.2	83.4	±12.1
Ambulatory BP, mmHg				
24-h systolic	127.3	±6.0	125.8	±5.1
24-h diastolic	76.2	±6.0	76.1	±5.8
Daytime systolic	129.6	±5.9	127.7	±5.3
Daytime diastolic	77.9	±5.9	77.8	±5.9
Nighttime systolic	114.9	±7.7	114.8	±5.9
Nighttime diastolic	65.7	±7.4	66.6	±6.7
Office BP, mmHg				
Systolic	116.5	±9.5	113.6	±10.2
Diastolic	68.9	±8.3	68.0	±8.6
Total cholesterol, mmol/L	5.14	±1.01	5.07	±0.89
Triglycerides, mmol/L	1.16	±0.53	1.05	±0.54
HDL cholesterol, mmol/L	1.74	±0.48	1.73	±0.41
LDL cholesterol, mmol/L	2.87	±0.84	2.85	±0.77
Cholesterol: HDL cholesterol ratio	3.11	±0.82	3.05	±0.82
Fasting glucose, mmol/L	4.98	±0.56	4.85	±0.49

^1^ Values are means ±SD unless otherwise stated. BMI, body mass index; BP, blood pressure; HDL, high-density lipoprotein; LDL, low-density lipoprotein.

**Table 2:** Anthropometric measures and cardiometabolic risk factors in adults with prehypertension supplemented for 6 weeks with a placebo (Plac) or a polyphenol extract and L-citrulline supplement (Suppl) (*n* = 69) ^1^.

	Plac ^1^		Suppl ^1^		Difference in Post-Treatment Values Suppl vs. Plac		
	Baseline	Post	Baseline	Post	Δ	Δ% ^2^	*p* ^3^
Body weight, kg	72.4 (2.5)	71.8 (0.2)	71.3 (2.4)	72.0 (0.2)	0.2 (0.2)	0.3	0.33
BMI, kg/m^2^	25.8 (0.7)	25.4 (0.1)	25.1 (0.7)	25.5 (0.1)	0.1 (0.1)	0.4	0.28
Waist circumference, cm	82.9 (1.1)	82.4 (0.3)	83.2 (1.1)	82.2 (0.3)	−0.2 (0.4)	−0.2	0.70
Ambulatory BP, mmHg							
24-h systolic	125.3 (1.1)	126.5 (0.8)	125.1 (1.1)	124.9 (0.8)	−1.6 (1.1)	−1.3	0.15
24-h diastolic	74.5 (1.1)	75.1 (0.6)	75.4 (1.1)	75.1 (0.5)	−0.1 (0.8)	−0.1	0.94
Daytime systolic	127.3 (1.1)	128.6 (0.8)	127.1 (1.1)	126.8 (0.8)	−1.8 (1.2)	−1.4	0.12
Daytime diastolic	76.4 (1.1)	77.3 (0.5)	77.1 (1.1)	76.8 (0.5)	−0.5 (0.8)	−0.6	0.53
Nighttime systolic	114.2 (1.4)	113.5 (0.9)	113.7 (1.4)	113.7 (0.9)	0.2 (1.3)	0.2	0.84
Nighttime diastolic	64.1 (1.3)	64.7 (0.7)	65.9 (1.3)	64.9 (0.7)	0.2 (1.0)	0.2	0.88
Office BP, mmHg							
Systolic	117.3 (1.9)	117.9 (1.0)	116.5 (1.9)	115.6 (1.0)	−2.3 (1.4)	−1.9	0.12
Diastolic	68.9 (1.5)	70.7 (0.9)	68.8 (1.5)	69.0 (0.9)	−1.7 (1.2)	−2.4	0.16
Total cholesterol, mmol/L	4.96 (0.12)	4.99 (0.08)	4.95 (0.12)	4.85 (0.08)	−0.15 (0.11)	−2.9	0.20
Triglycerides ^4^, mmol/L	1.03 (0.09)	1.03 (0.05)	0.89 (0.09)	0.97 (0.05)	−0.07 (0.07)	−6.4	0.30
HDL cholesterol, mmol/L	1.67 (0.07)	1.71 (0.03)	1.75 (0.07)	1.68 (0.03)	−0.03 (0.04)	−1.8	0.49
LDL cholesterol, mmol/L	2.76 (0.13)	2.76 (0.07)	2.74 (0.12)	2.68 (0.07)	−0.09 (0.09)	−3.1	0.36
Cholesterol: HDL cholesterol ratio	3.17 (0.14)	3.08 (0.06)	3.00 (0.14)	2.99 (0.06)	−0.09 (0.08)	−2.8	0.29
hs-CRP ^4^, mg/L	0.76 (0.21)	0.73 (0.13)	0.73 (0.20)	0.86 (0.13)	0.12 (0.19)	17.0	0.36
AGEs, AU	199 (8)	218 (8)	203 (8)	199 (8)	−20 (11)	−9.1	0.07

^1^ Values are least-squares means (SEM) unless otherwise stated. For all variables: Plac, *n* = 34 and Suppl, *n* = 35 (except for nighttime BP in Suppl group, *n* = 34, and for hs-CRP in Plac group, *n* = 32). AGEs, advanced glycation end products; BP, blood pressure; AU, arbitrary units; hs-CRP, high-sensitivity C-reactive protein. ^2^ The % values are shown to give an idea of the effect size (difference in outcome between the supplement and the placebo).^3^ *p* values were for the main treatment effects comparing post-treatment values between Plac and Suppl in the GENMOD (generalized linear model) procedure. Covariates (baseline values of the selected variable, sex, age, body weight, waist circumference, physical activity and total energy expenditure during the ambulatory BP measurement) were included in the model only when they were significant.^4^ Analyses were performed on log-transformed data because of the skewness of distributions of the model residues. Data are presented as geometric means and geometric SEM factor expressed as a ratio relative to the geometric means.

## Data Availability

The data that support the findings of this study are available upon request from the authors.

## Supplementary Materials

The following are available online at https://www.mdpi.com/2072-6643/13/2/399/s1, Table S1. Prevalence of medication and supplement use in the placebo (Plac) and polyphenol extract/L-citrulline supplement (Suppl) group throughout the 6-week intervention; Table S2. List of participants under stable medication and change in medication during the intervention in the placebo (Plac) and polyphenol extract/L-citrulline supplement (Suppl) group.
