# Supplementary material for: Sex May Modulate the Effects of Combined Polyphenol Extract and L-citrulline Supplementation on Ambulatory Blood Pressure in Adults with Prehypertension: A Randomized Controlled Trial
Source: Nutrients. 2021 Jan 27;13(2):399. doi: 10.3390/nu13020399 (PMC7912201; doi:10.3390/nu13020399)
Supplement: Supplementary file 1 [file nutrients-13-00399-s001.pdf]

Sex may modulate the effects of combined polyphenol extract and L-citrulline supplementation on ambulatory blood pressure in adults with pre-hypertension: a randomized controlled trial. Vors *et al.* Online Supplementary material

Table 1. Prevalence of medication and supplement use in the placebo (Plac) and polyphenol extract/L-citrulline supplement (Suppl) group throughout the 6-week intervention.

	**Plac**	**Suppl**
Total *n*	34	35
Medication users, *n* (%)	23 (67.7%)	24 (68.6%)
NHP users, *n* (%)	18 (52.9%)	23 (65.7%)
Medication and NHP users, *n* (%)	16 (47.1%)	17 (48.6%)
Change in medication, *n* (%)	4 (11.8%)	7 (20.0%)
Change in NHP, *n* (%)	3 (8.8%)	2 (5.7%)

NHP: Natural Health Product.

Sex may modulate the effects of combined polyphenol extract and L-citrulline supplementation on ambulatory blood pressure in adults with pre-hypertension: a randomized controlled trial. Vors *et al.* Online Supplementary material

Sex may modulate the effects of combined polyphenol extract and L-citrulline supplementation on ambulatory blood pressure in adults with pre-hypertension: a randomized controlled trial. Vors *et al.* Online Supplementary material

Table S2. List of participants under stable medication and change in medication during the intervention in Table . he placebo (Plac) and polyphenol extract/L-citrulline supplement (Suppl) group.

Participant			Plac		
ID	Medication 1	Medication 2	Medication 3	Medication 4	Medication 5
5	Citalopram 20 mg QD	Levonorgestrel IUD			
6	Estradiol 1 tablet QD				
8	Acetaminophen PRN				
9	Levothyroxine 125 mcg QD				
11	Ethinyl Estradiol/Levonorgestrel 1 tablet QD				
14	Levothyroxine 100 mcg QD				
25	Acetaminophen PRN				
35	Ethinyl Estradiol/Levonorgestrel 1 tablet QD	Lorazepam PRN	Flu vaccine [3]		
51	Risedronate 35 mg 1x/wk				
58	Estradiol gel QD	Progesterone QD			
61	Citalopram QD	Risedronate 35 mg 1x/wk			
71	Aripiprazole 2 mg QD	Sertraline 50 mg QD	Levonorgestrel IUD	Fluticasone Furoate nasal PRN [1]	
97	Acetaminophen PRN				
106	Sertraline 200 mg QD	Levonorgestrel IUD	Acetaminophen PRN		
108	Acetaminophen PRN				
122	Conjugated Estrogens 2x/wk	Acetaminophen PRN			
133	Levothyroxine 50 mcg QD	Acetaminophen PRN			
163	Alfuzosin 10 mg QD	Acetaminophen PRN			
167	Cetirizine 10 mg PRN				
169	Citalopram 10 mg 1x/2 d				
170	Levothyroxine 50 mcg QD				
173	Nitrofurantoin 1x/2 d				
175	Medroxyprogesterone 2.5 mg QD	Estradiol vaginal	Esomeprazole PRN	Desloratadine PRN [1]	
182	Cetirizine PRN [1]				

Participant			Suppl		
ID	Medication 1	Medication 2	Medication 3	Medication 4	Medication 5
7	Antacid PRN	Acetaminophen PRN			
15	Isotretinoin 10 mg 1x/wk	Oflaxacin opht. [1, 2]	Acetaminophen PRN		
16	Ibuprofen [3]				
27	Acetaminophen PRN				
30	Eletriptan 25 mg PRN	Acetaminophen PRN	Fluticasone Propionate 250 mcg QD [1]	Clarithromycin 500 mg QD [1]	Albuterol 100 mcg QD [1]
32	Methylphenidate 80 mg QD	ZolpQDem 5 mg 1x/2 d	Acetaminophen PRN		
34	Allopurinol 2 mg QD				
76	Ethinyl Estradiol/Levonorgestrel 1 tablet QD				
77	Estradiol gel QD	Progesterone QD	Acetaminophen PRN		
78	Acetaminophen PRN				
87	Fluticasone Furoate 2 inhal. QD	Levonorgestrel 1 tablet QD	Acetaminophen PRN		
102	Ethinyl Estradiol/Levonorgestrel 1 tablet QD	Albuterol PRN			
110	Mometasone Furoate nasal QD 2	Budesonide 2 inhal. BID [2]	Albuterol PRN [2]	Acetaminophen PRN	
112	Desogestrel/Ethinyl Estradiol 1 tablet QD	Acetaminophen PRN			
113	Ethinyl Estradiol/Levonorgestrel 1 tablet QD	Desloratadine PRN	Acetaminophen PRN		
120	Bromazepam 1.5 mg PRN	Acetaminophen PRN			
129	Acetaminophen PRN				
139	Acetaminophen PRN				
147	Ethinyl Estradiol/Ethynodiol Diacetate 1 tablet QD	Acetaminophen PRN			
158	Acetaminophen PRN	Nasonex PRN [1]			
160	Valacyclovir PRN	Acetaminophen PRN			
165	Fluorometholone opht. BID	Timolol opht. BID			
172	Atomoxetine 25 mg QD	Levonorgestrel IUD	Metronidazole QD [1]		
180	Acetaminophen PRN				
181	Acetaminophen PRN				
184	Cetirizine PRN [1]				

Shaded grey cells indicate change during the study: [1] Initiated during the course of the study, [2] Stopped during the study, [3] Unique dose, 10 days prior to testing

BID: twice-daily; inhal.: inhalation; IUD: intrauterine device; opht.: ophthalmic; PRN: "pro re nata", which means "as needed"; QD: once-daily
